# Precision-weighted mixed models identify high-yielding and temporally stable cowpea germplasm across three growing seasons

**DOI:** 10.3389/fpls.2026.1926051

**Published:** 2026-10-07

**Authors:** Sami Jan, M. Ashraf Bhat, Akshay Kumar, Nakeeb-un- Nisa, M. Altaf Wani, Farooq Ahmad Bhat, Phalisteen Sultan, Zafir Ahmad Naik

**Affiliations:** 1 Division of Genetics and Plant Breeding, Faculty of Agriculture (FoA), Sher-e-Kashmir University of Agricultural Sciences and Technology of Kashmir (SKUAST-K), Wadura, J&K, India; 2 Division of Plant Pathology, Faculty of Agriculture (FoA), Sher-e-Kashmir University of Agricultural Sciences and Technology of Kashmir (SKUAST-K), Wadura, J&K, India; 3 Plant Molecular Biology and Biotechnology, Council of Scientific and Industrial Research–Indian Institute of Integrative Medicine (CSIR–IIIM), Srinagar, J&K, India

**Keywords:** augmented block design, genotype × year interaction, REML-BLUP, temporal stability, *Vigna unguiculata*, WAASBY

## Abstract

**Introduction:**

Cowpea is an important multipurpose legume that contributes to food and nutritional security, particularly in regions affected by environmental stress. Identifying high-yielding and stable germplasm is essential for improving breeding efficiency and developing resilient cultivars. However, distinguishing persistent genetic merit from seasonal environmental noise remains challenging when trial precision varies among years.

**Methods:**

Evaluated 150 International Institute of Tropical Agriculture cowpea germplasm lines and four repeated checks over three growing seasons (2020–2022) at a temperate site in Kashmir, India, using augmented block trials. Restricted maximum likelihood and best linear unbiased prediction models accounted for genotype × year interaction and year-specific residual variances.

**Results:**

Seed yield per plant showed high genetic variability (GCV 59.75%), Cullis heritability of 0.991, and prediction accuracy of 0.992. The genotype × year-to-genotype variance ratio was 0.017, while across-year genetic correlations for yield were 0.980–0.998, indicating strong preservation of genotype rankings. Residual variance was markedly larger in 2022, and modeling heterogeneous residuals improved AIC by 50.29. IITA-122 had the highest predicted yield (156.94 g plant^-1^), 100.60% above the best check, whereas IITA-84 had the best productivity–stability profile (WAASBY 93.85; WAASB 1.61). Thirteen genotypes met an integrated high-confidence selection rule.

**Discussion:**

The selected lines demonstrated persistent genetic merit and temporal stability despite differences in trial precision among years. They warrant replicated, multi-location validation because the present study demonstrates temporal stability at a single location rather than broad adaptation.

## Introduction

Cowpea [*Vigna unguiculata* (L.) Walp.] is a multipurpose grain legume with an important role in food and nutritional security across tropical and subtropical production systems. Its protein-rich grain, ability to fix atmospheric nitrogen, short crop duration, and relative tolerance of heat, intermittent drought, and low-input conditions make it especially useful where higher productivity must be achieved without a comparable increase in external inputs (Carvalho et al., 2017; Boukar et al., 2019). Yet yields remain constrained by biotic stress, soil limitations, climatic variability, and the limited availability of locally adapted, high-yielding cultivars (Boukar et al., 2019). Genetic diversity is central to addressing these constraints. The IITA cowpea collection contains broad variation, and molecular studies of global germplasm have revealed substantial population structure and allelic diversity that can be exploited in breeding (Fatokun et al., 2018). Genome-scale studies have since identified loci associated with agronomic and quality traits, further supporting the integration of diverse germplasm with modern prediction and selection approaches (Wu et al., 2024). The continued genetic gains reported from the IITA breeding program also suggest that cowpea yield has not yet reached a biological plateau (Ongom et al., 2024). Performance in a single season is not necessarily a reliable measure of lasting genetic merit. Yield is a complex quantitative trait shaped by genotype, environment, and their interaction. Genotype × environment interaction becomes especially consequential when genotypes change rank across environments, because re-ranking directly reduces selection efficiency. Its importance therefore depends not only on the magnitude of interaction variance, but also on its size relative to persistent genotypic variance and on the genetic correlations among environments (Smith et al., 2005; Malosetti et al., 2013). Multi-year testing at one site addresses a narrower but important question: whether genotype rankings persist through temporal environmental variation. It does not, by itself, demonstrate broad geographic adaptation.

Linear mixed models are particularly useful for augmented and other unbalanced breeding trials. They allow variance components to be estimated by REML, genetic merit to be predicted by BLUP, and uncertainty to be carried with each prediction instead of assuming that all adjusted means are equally precise (Cullis et al., 2006; Piepho et al., 2008). That distinction becomes important when experimental precision differs among seasons. Heterogeneous residual variance changes the effective contribution of each year, while covariance models can help separate simple changes in scale from genuine crossover interaction (Edwards and Jannink, 2006; Gogel et al., 2018). Under these conditions, prediction-error-based measures such as Cullis heritability are better suited than formulas that assume balanced replication and homogeneous precision (Piepho and Möhring, 2007; Schmidt et al., 2019).

Breeding decisions must also consider whether high performance is accompanied by acceptable stability. WAASB summarizes the magnitude of BLUP-based interaction effects, whereas WAASBY combines performance and stability under an explicit breeder-defined weighting scheme (Olivoto et al., 2019a; Olivoto et al., 2019b). Used alongside prediction accuracy and selection probability, these measures can distinguish genotypes that are not only productive, but also well resolved statistically and consistent across seasons. Against this background, we evaluated 150 IITA cowpea germplasm lines and four repeated checks over three growing seasons under temperate conditions in Kashmir. Our objectives were to (i) quantify year-wise genetic differentiation and field precision; (ii) partition variation into genotypic, genotype × year (G×Y), and residual components; (iii) estimate GCV, PCV, Cullis heritability, BLUP accuracy, and expected genetic advance; (iv) test and model residual heterogeneity; (v) quantify across-year genetic correlations and rank preservation; and (vi) identify an elite set that combines predicted yield, temporal stability, and selection certainty for subsequent replicated multi-location testing.

## Results

### Year-wise genetic differentiation and experimental precision

The year-wise augmented-block analyses consistently detected genotypic differences across all nine traits. Genotype effects were significant at P < 0.001 in each of the 2020, 2021, and 2022 seasons (**Table 1**), with particularly strong differentiation for PH, NPPP, and SY. For SY, genotype F statistics were 199.93 in 2020, 266.63 in 2021, and 11.37 in 2022. The much smaller value in 2022 coincided with a sharp rise in residual dispersion: error CV increased to 17.27%, compared with 4.14% in 2020 and 3.60% in 2021. Error CVs for most other traits remained below 10% within individual years, suggesting generally good field precision.

**Table 1:** Year-wise augmented-block genotype tests and experimental precision for nine cowpea traits.

Trait	2020 F (P)	2021 F (P)	2022 F (P)	CV 2020 (%)	CV 2021 (%)	CV 2022 (%)
DTF	35.64 (1.66 × 10^-^¹^6^)	227.98 (3.09 × 10^-^²^7^)	276.80 (2.28 × 10^-^²^8^)	3.51	1.46	1.30
DTM	3.01 (6.91 × 10^-4^)	13.03 (6.45 × 10^-^¹¹)	21.97 (8.80 × 10^-^¹^4^)	2.88	2.41	1.54
PH	337.95 (1.55 × 10^-^²^9^)	492.19 (9.84 × 10^-^³²)	337.68 (1.57 × 10^-^²^9^)	2.49	1.97	2.45
NPBPP	16.30 (3.93 × 10^-^¹²)	15.83 (5.68 × 10^-^¹²)	13.68 (3.53 × 10^-^¹¹)	6.36	7.69	9.00
NPPP	64.39 (6.78 × 10^-^²^0^)	120.46 (1.60 × 10^-^²³)	106.93 (7.89 × 10^-^²³)	4.79	3.45	3.90
PL	28.96 (2.48 × 10^-^¹^5^)	44.16 (9.93 × 10^-^¹^8^)	40.07 (3.57 × 10^-^¹^7^)	5.15	4.30	4.71
NSPP	7.35 (6.24 × 10^-8^)	10.70 (7.24 × 10^-^¹^0^)	13.46 (4.31 × 10^-^¹¹)	8.33	6.96	5.63
SW	16.54 (3.27 × 10^-^¹²)	19.57 (3.87 × 10^-^¹³)	19.21 (4.90 × 10^-^¹³)	5.29	5.89	6.15
SY	199.93 (1.80 × 10^-^²^6^)	266.63 (3.77 × 10^-^²^8^)	11.37 (3.45 × 10^-^¹^0^)	4.14	3.60	17.27

F tests used 153 numerator and 27 denominator degrees of freedom. Exact P values were calculated from the reported F statistics. CV, residual coefficient of variation.

The combined fixed-effect ANOVA for SY reinforced this pattern. Year was significant (F = 7.11, P = 0.0014), and the genotype effect was highly significant (F = 94.63, P = 9.71 × 10^-6^¹). In contrast, the G×Y mean-square test was not significant (F = 0.994, P = 0.528; **Supplementary Table S2**). At the aggregate mean-square level, there was therefore no evidence of pronounced crossover interaction for seed yield.

### REML variance architecture, genetic variability and predicted selection potential

REML estimates showed that persistent genotypic variation accounted for most of the modeled variance in several traits, although the balance varied across the phenotypic panel (**Table 2**; **Figure 1**). The G×Y-to-genotype variance ratio was lowest for SY (0.017), DTF (0.023), SW (0.023), NPPP (0.034), and PH (0.049). At the other end of the spectrum, NSPP had the largest ratio (0.462), followed by PL (0.174), DTM (0.160), and NPBPP (0.152). Temporal repeatability was therefore strongly trait dependent.

**Table 2:** REML variance components, genetic variability, prediction reliability, and expected selection response.

*Trait*	*Mean*	*σ²G*	*σ²G×Y*	*G×Y/G*	*GCV %*	*PCV %*	*Cullis H²*	*BLUP accuracy*	*GAM %*	*Consistency*
*DTF*	*84.66*	*94.94*	*2.20*	*0.023*	*11.51*	*11.62*	*0.984*	*0.989*	*23.52*	*0.977*
*DTM*	*113.10*	*53.52*	*8.55*	*0.160*	*6.47*	*6.80*	*0.904*	*0.948*	*12.68*	*0.862*
*PH*	*98.17*	*1768.74*	*86.72*	*0.049*	*42.84*	*43.21*	*0.983*	*0.988*	*87.63*	*0.953*
*NPBPP*	*10.54*	*8.93*	*1.36*	*0.152*	*28.35*	*29.46*	*0.922*	*0.957*	*56.29*	*0.868*
*NPPP*	*28.42*	*115.20*	*3.91*	*0.034*	*37.77*	*38.07*	*0.984*	*0.989*	*77.30*	*0.967*
*PL*	*18.24*	*20.11*	*3.49*	*0.174*	*24.58*	*25.43*	*0.932*	*0.962*	*49.01*	*0.852*
*NSPP*	*12.28*	*4.08*	*1.88*	*0.462*	*16.44*	*18.11*	*0.818*	*0.901*	*30.79*	*0.684*
*SW*	*14.99*	*12.10*	*0.28*	*0.023*	*23.21*	*23.58*	*0.964*	*0.979*	*47.12*	*0.977*
*SY*	*63.64*	*1445.66*	*24.59*	*0.017*	*59.75*	*60.08*	*0.991*	*0.992*	*122.59*	*0.983*

G×Y/G = σ²G×Y/σ²G. Consistency = σ²G/(σ²G + σ²G×Y). GAM is expected genetic advance as a percentage of the mean at 5% selection intensity.\.

**Figure 1:**
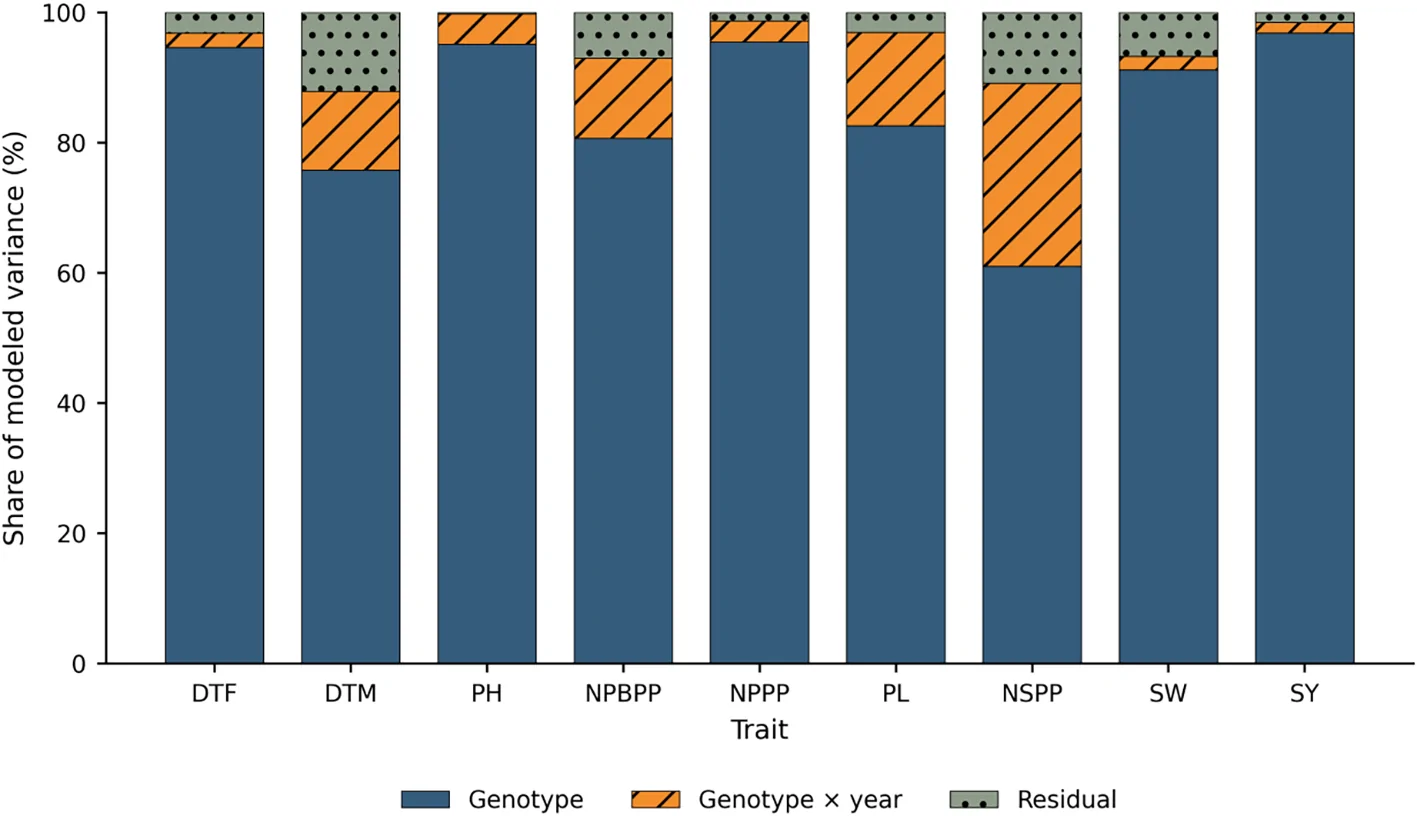
Relative partitioning of modeled variance across cowpea traits. Genotypic, genotype × year, and residual contributions are shown for nine agronomic and yield-related traits across three growing seasons. Proportional contributions are displayed because traits are measured in different units.

SY showed by far the greatest relative genetic dispersion. GCV was 59.75% and PCV was 60.08%, differing by only 0.33 percentage points. The broad spread among genotypes was therefore not mainly attributable to residual variation. PH (GCV = 42.84%; PCV = 43.21%) and NPPP (37.77%; 38.07%) were also highly variable, whereas DTM had much less relative dispersion (6.47%; 6.80%).

Reliability based on prediction error was high for the main selection traits. Cullis heritability was 0.991 for SY, 0.984 for DTF, 0.984 for NPPP, 0.983 for PH, and 0.964 for SW, with corresponding mean BLUP accuracies between 0.979 and 0.992. NSPP was the least reliable trait (Cullis H² = 0.818; accuracy = 0.901), consistent with its larger G×Y component. Predicted GAM was highest for SY (122.59%), followed by PH (87.63%), NPPP (77.30%), and NPBPP (56.29%) (**Supplementary Figure S1**).

### Heterogeneous residual variance and model adequacy

A common residual variance across years was inadequate for several traits, most notably SY. The Brown–Forsythe test for SY was significant (F = 5.25, P = 0.0065), and allowing year-specific residual variances reduced AIC from 4339.20 to 4288.90 (ΔAIC = 50.29; likelihood-ratio P = 1.62 × 10^-^¹²; **Supplementary Table S1**). The estimated residual variances were 7.92 in 2020, 5.90 in 2021, and 56.55 in 2022 (**Supplementary Figure S4**). Residual variance in 2022 was therefore about 7.1 times that of 2020 and 9.6 times that of 2021. Heterogeneous residual structures were also selected for DTF, DTM, and NPPP; homogeneous structures were adequate for PH, NPBPP, PL, NSPP, and SW.

### Across-year genetic covariance and preservation of genotype ranking

Across-year genetic correlations made the degree of temporal rank preservation clear (**Supplementary Table S1**; **Figure 2**). For SY, correlations were 0.980 for 2020–2021, 0.991 for 2020–2022, and 0.998 for 2021–2022. The unstructured covariance model improved AIC by 18.92 units relative to compound symmetry, yet all three correlations remained close to one. DTF, SW, and NPPP showed similarly strong continuity, with pairwise correlations above 0.962 in every case. PH was also highly repeatable, with correlations ranging from 0.924 to 0.973.

**Figure 2:**
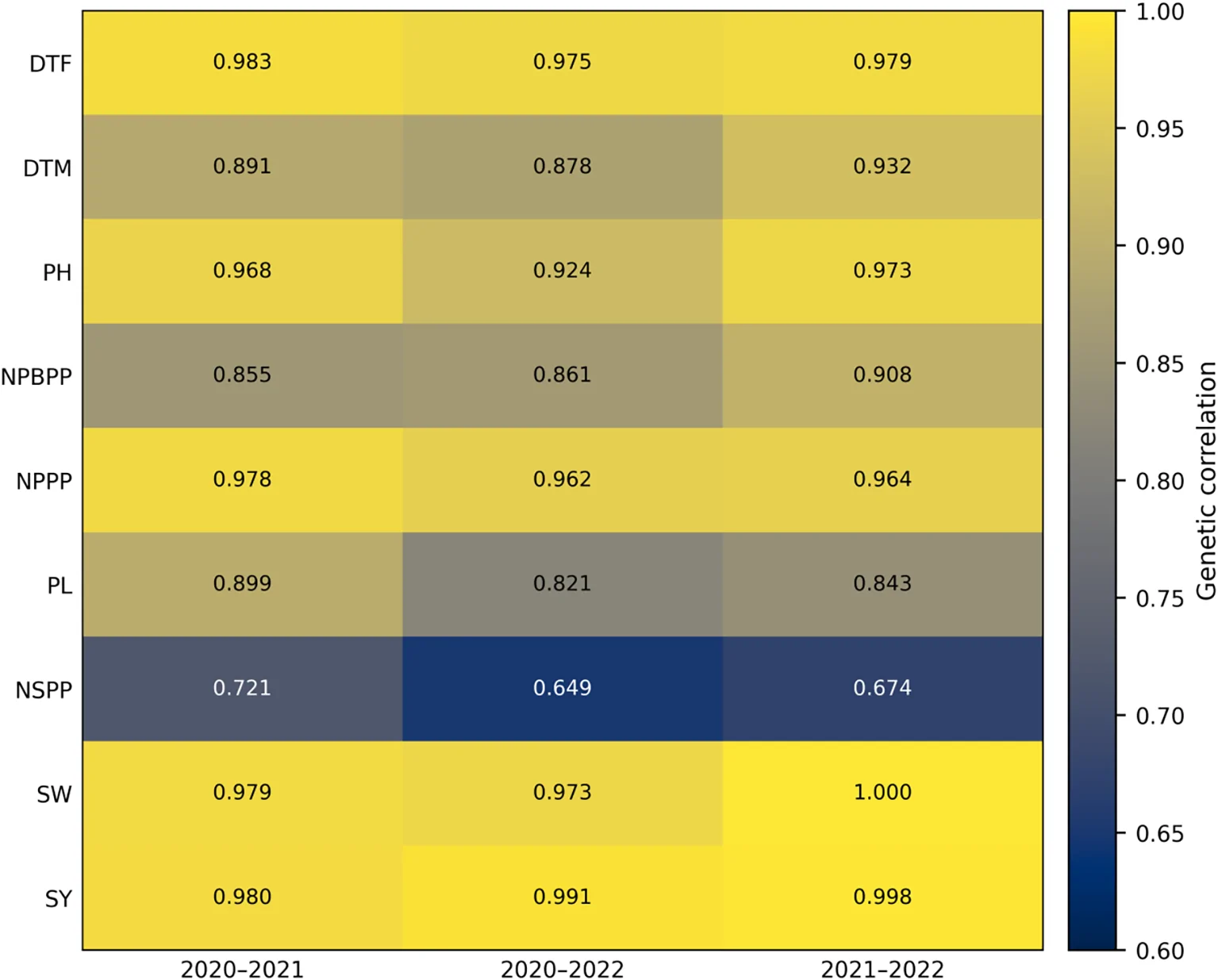
Genetic correlations among growing seasons. Pairwise model-based genetic correlations for 2020–2021, 2020–2022, and 2021–2022 quantify preservation of genotype ranking across years. Seed yield, 100-seed weight, days to 50% flowering, and pod number show high temporal continuity, whereas seeds per pod is less repeatable.

NSPP was much less stable over time: its genetic correlations were 0.721, 0.649, and 0.674, consistent with the large σ²G×Y/σ²G ratio for this trait. PL (0.821–0.899), NPBPP (0.855–0.908), and DTM (0.878–0.932) showed intermediate consistency. In other words, seed yield was unusually robust across seasons, while seed number per pod was far more sensitive to year-dependent expression.

### Mixed-model prediction identifies a distinct high-yielding group

The high reliability of the fitted model clearly separated the leading seed-yield genotypes (**Table 3**; **Figure 3**). IITA-122 had the highest overall adjusted predicted yield at 156.94 g plant^-^¹ (95% PI 149.32–164.57), followed closely by IITA-246 (156.20), IITA-153 (155.21), IITA-52 (155.09), and IITA-270 (154.91). Prediction accuracy for each of these leading entries was approximately 0.992. SKUA-WCP-149 was the strongest repeated check, with an overall predicted SY of 78.24 g plant^-^¹ and an accuracy of 0.994 (**Supplementary Table S3**).

**Table 3:** High-confidence elite cowpea genotypes and the best repeated check.

Genotype	Overall SY	95% PI	WAASB	WAASBY	Yield rank	Superiority vs best check (%)	Top-10% probability
IITA-84	150.56	142.94–158.18	1.61	93.85	11	92.44	1.000
IITA-122	156.94	149.32–164.57	3.59	92.19	1	100.60	1.000
IITA-95	152.02	144.39–159.64	2.87	91.61	8	94.30	1.000
IITA-257	149.95	142.32–157.57	2.65	91.16	12	91.66	1.000
IITA-264	150.74	143.11–158.36	3.14	90.40	10	92.67	1.000
IITA-153	155.21	147.58–162.83	4.15	90.10	3	98.38	1.000
IITA-246	156.20	148.57–163.82	5.56	87.29	2	99.64	1.000
IITA-270	154.91	147.29–162.53	5.31	87.29	5	98.00	1.000
IITA-203	142.42	134.80–150.04	3.46	85.87	15	82.04	0.954
IITA-174	152.11	144.49–159.73	5.94	84.55	7	94.42	1.000
IITA-77	147.63	140.00–155.25	5.52	83.48	13	88.69	1.000
IITA-191	143.74	136.12–151.37	5.08	82.73	14	83.73	0.982
IITA-52	155.09	147.46–162.71	7.65	81.97	4	98.23	1.000
SKUA-WCP-149	78.24	71.98–84.49	1.88	60.22	37	0.00	0.000

Elite status required top-decile overall predicted seed yield, top-decile WAASBY, and conditional probability ≥0.80 of belonging to the yield top 10%. WAASBY uses 65% weight on performance and 35% on stability. SY is g plant^-^¹. Superiority is relative to SKUA-WCP-149.

**Figure 3:**
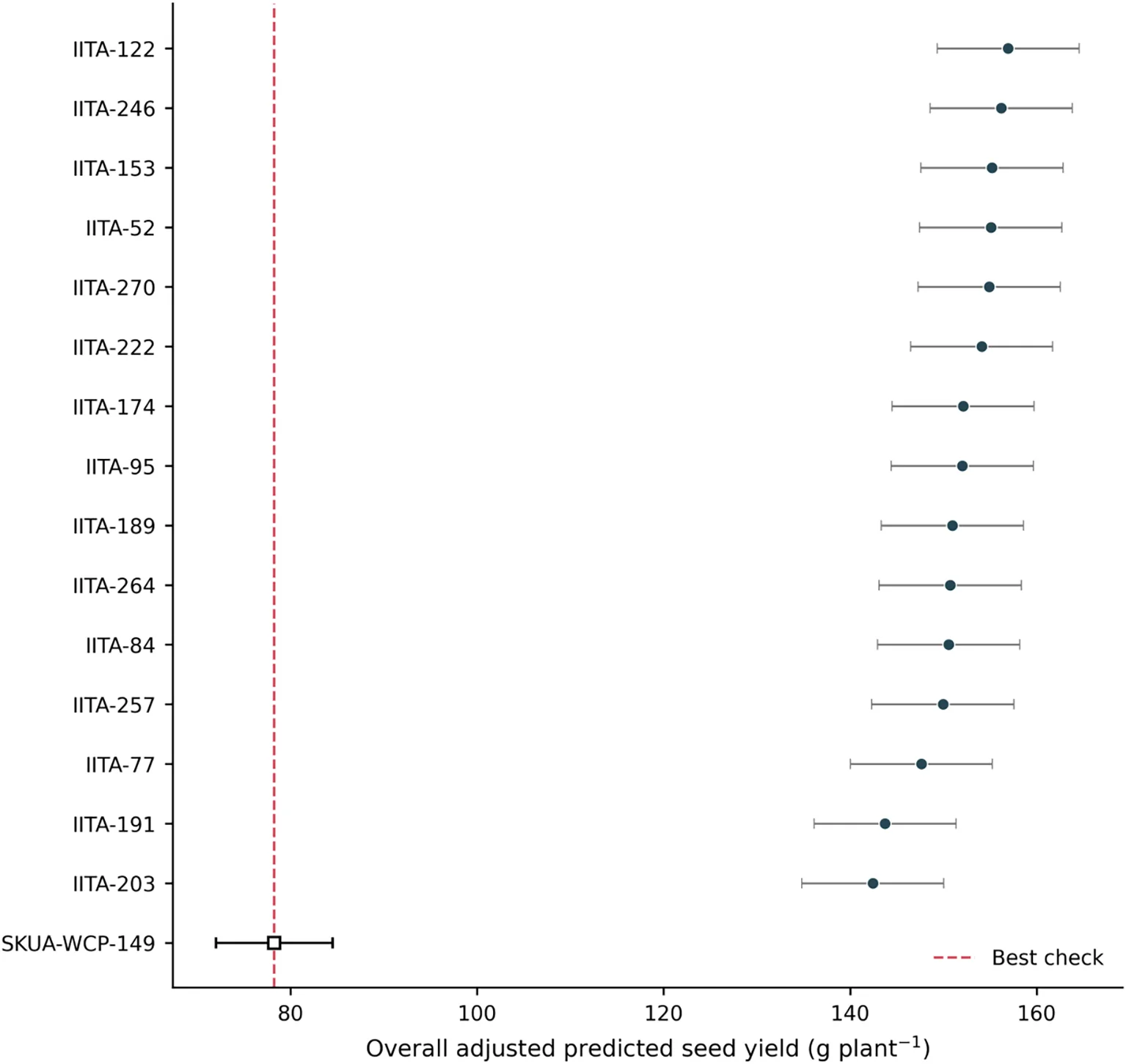
Predicted seed yield of leading cowpea genotypes. Overall adjusted predicted seed yield is shown for the 15 highest-yielding test genotypes and the best repeated check, with 95% prediction intervals. The dashed line denotes the predicted mean of SKUA-WCP-149, the best repeated check.

Relative to SKUA-WCP-149, the predicted advantage was 100.60% for IITA-122, 99.64% for IITA-246, and 98.38% for IITA-153. The prediction intervals of the leading test genotypes were clearly separated from that of the best check, so their estimated superiority was large relative to prediction uncertainty. This supports advancement to further replicated testing, but not cultivar release.

### Reaction norms and the productivity–stability trade-off

The leading genotypes followed broadly parallel reaction norms across 2020–2022 and remained well above the best check (**Figure 4**). IITA-122 rose from 154.59 g plant^-^¹ in 2020 to 162.62 in 2021, then returned to 153.62 in 2022. IITA-84 varied less, moving from 149.26 to 154.13 and then to 148.29. -Seasonal responses among elite lines thus differed more in magnitude than in rank order, in keeping with the small SY interaction variance and near-unity genetic correlations.

**Figure 4:**
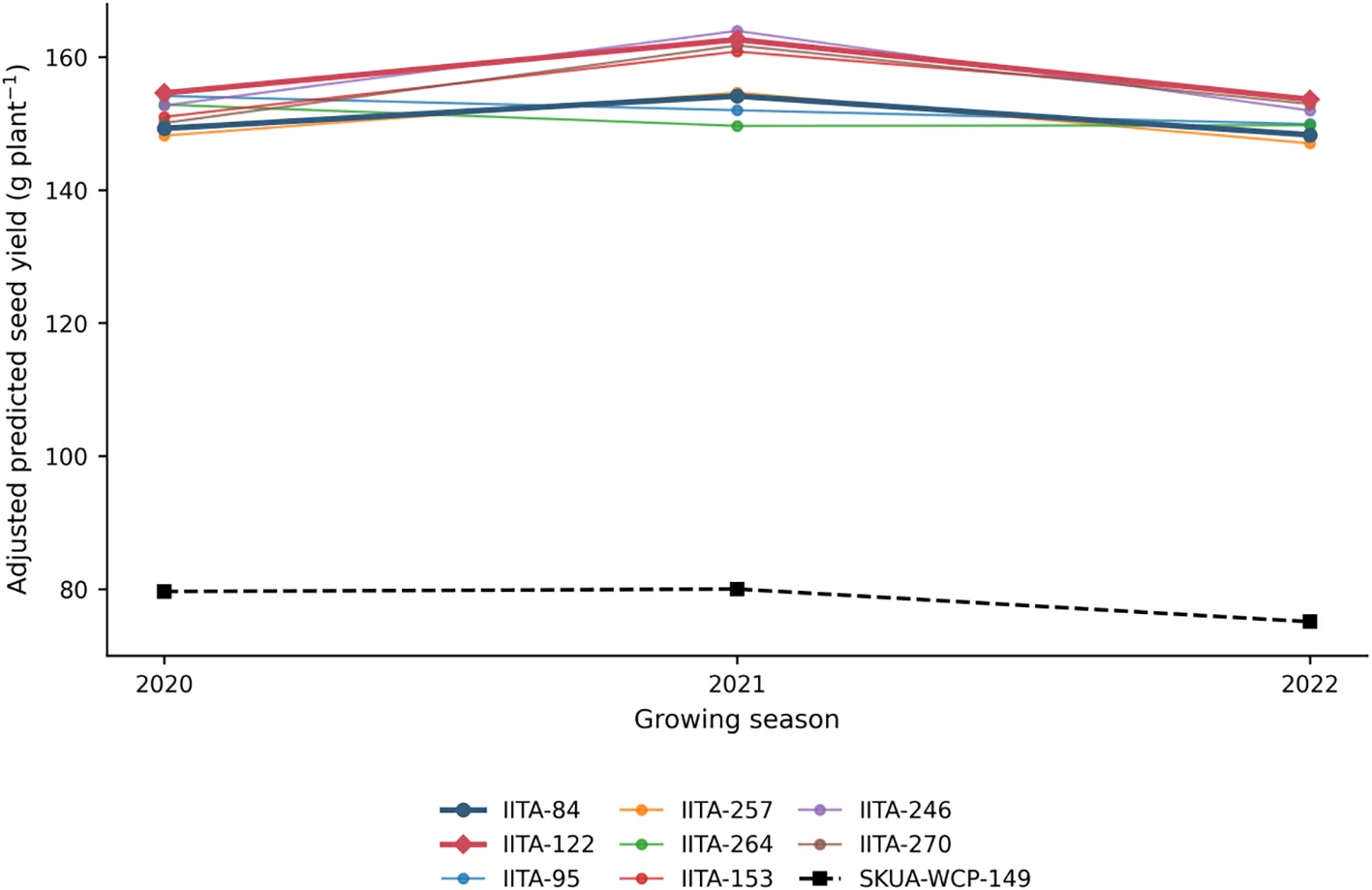
Temporal reaction norms of leading genotypes. Year-specific adjusted seed-yield predictions are shown for leading test genotypes and SKUA-WCP-149 across 2020–2022. Nearly parallel trajectories among elite lines indicate limited crossover interaction for seed yield.

Stability analysis made clear that maximum mean yield and the best breeding recommendation were not necessarily the same. IITA-84 combined a high overall predicted SY (150.56) with a low WAASB of 1.61. IITA-122 ranked first for mean yield but had a larger WAASB of 3.59. IITA-95 (2.87) and IITA-257 (2.65) also occupied favorable positions, combining high yield with relatively small interaction deviations. IITA-52, by comparison, had very high predicted yield (155.09) but was less stable (WAASB = 7.65) (**Figure 5**).

**Figure 5:**
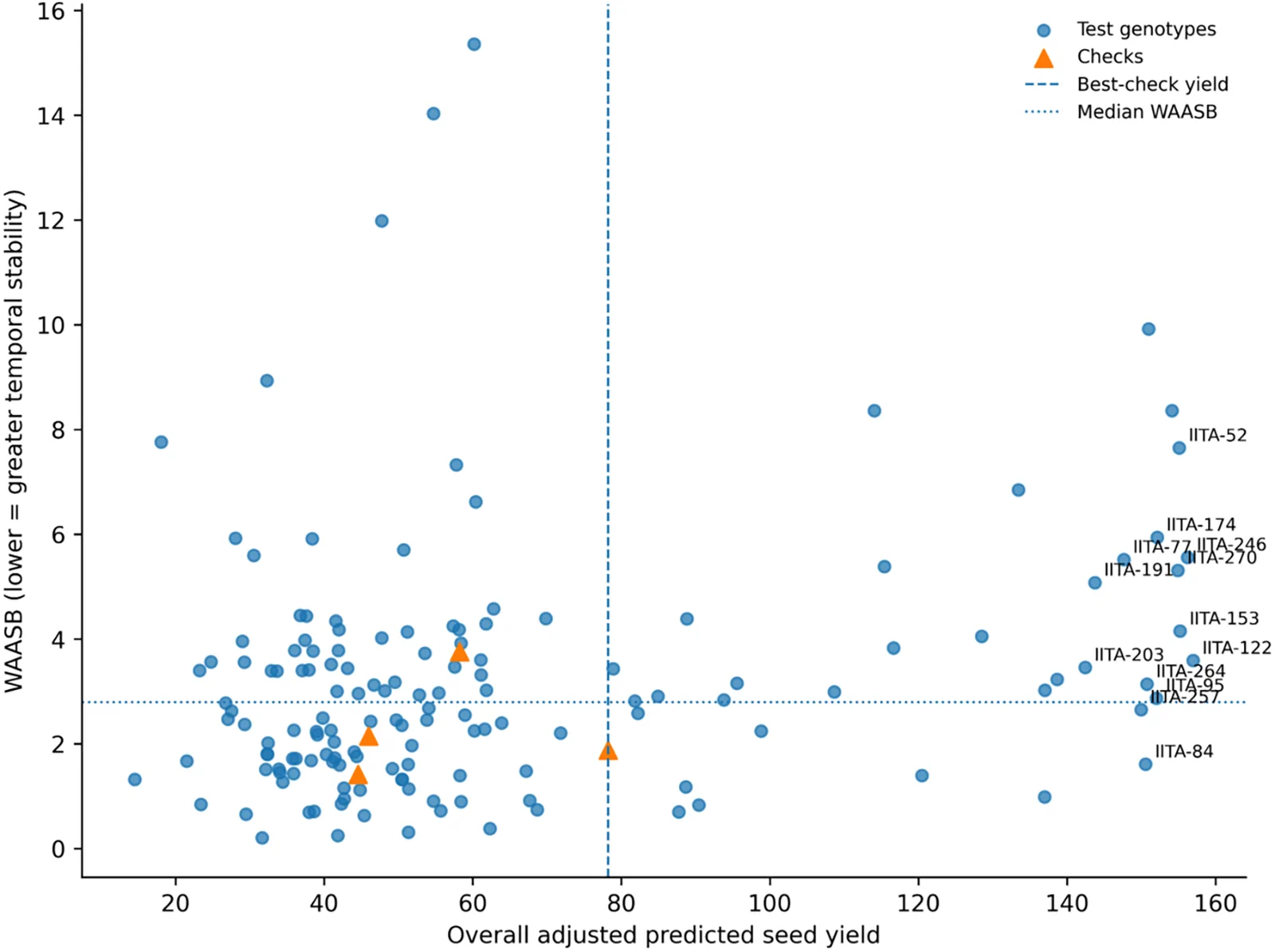
Productivity–stability landscape for seed yield. Overall adjusted predicted seed yield is plotted against WAASB, where lower WAASB indicates greater temporal stability. The reference lines mark the best-check yield and median WAASB; the most favorable region combines high predicted yield with low interaction deviation.

Under the 65:35 performance-to-stability weighting, WAASBY ranked IITA-84 first (93.85), followed by IITA-122 (92.19), IITA-95 (91.61), IITA-257 (91.16), and IITA-264 (90.40) (**Supplementary Figure S2**). SKUA-WCP-149, the best repeated check, was relatively stable (WAASB = 1.88) but much less productive (predicted SY = 78.24), which illustrates why stability alone is not a useful selection target. Mean SY was almost perfectly aligned with HMGV, RPGV, and HMRPGV (Spearman r = 0.9997–1.0000). WAASBY remained strongly, though not perfectly, correlated with mean SY (r = 0.902), while stability alone (−WAASB) was negatively correlated with mean SY (r = −0.213) and only weakly related to WAASBY (r = 0.120; **Supplementary Figure S2**).

### Integrated elite selection and predicted selection response

Thirteen genotypes satisfied all three high-confidence selection criteria: IITA-84, IITA-122, IITA-95, IITA-257, IITA-264, IITA-153, IITA-246, IITA-270, IITA-203, IITA-174, IITA-77, IITA-191, and IITA-52 (**Table 3**). The decision heatmap (**Supplementary Figure S3**) shows that they reached this status through different combinations of productivity and stability. IITA-84 had the most balanced profile. IITA-122 led for yield and superiority over the best check but occupied only a moderate stability percentile, whereas IITA-52 entered the elite set largely because of its high yield despite its low stability percentile.

The top-decile WAASBY group contained 16 entries with a predicted mean SY of 148.76 g plant^-^¹, compared with 63.64 g plant^-^¹ for the full population (**Supplementary Table S4**). This corresponds to a predicted selection differential of 85.12 g plant^-^¹, or 133.76% of the population mean; the selected-group mean was also 90.14% above the best check. Fourteen genotypes were common to the top-decile yield and top-decile WAASBY groups, and 13 remained after the ≥0.80 conditional selection-probability requirement was applied. These values are model-based selection differentials, not realized genetic gains.

## Discussion

The most important point from the three-year trial is that the observed diversity was not a one-season effect. Genotypic differences recurred across all nine traits and remained estimable with high precision after accounting for the augmented design. Augmented trials allow large numbers of unreplicated entries to be screened while repeated checks provide the experimental-error basis needed for adjustment (Federer, 1961). Mixed models extend that framework by accommodating unequal precision and correlated effects and by predicting genetic merit rather than treating every adjusted mean as equally reliable (Smith et al., 2005; Cullis et al., 2006). For complex, unbalanced trial series, one-stage or appropriately weighted mixed models are generally preferred because they retain plot-level information and model non-genetic heterogeneity directly (Gogel et al., 2018). Viewed in that context, the large genotype F statistics and generally low error CVs point to genuine, repeatable phenotypic differences within the panel.

The 2022 SY results also illustrate why statistical significance alone is a poor measure of trial quality. Genotype differences were still highly significant, yet error CV rose to 17.27% and residual variance was almost ten times the 2021 estimate. Ignoring that heterogeneity can distort variance components, alter genotype rankings, and give a low-precision environment too much influence in a pooled analysis (Edwards and Jannink, 2006). Allowing a separate residual variance for each year meant that the REML model effectively weighted seasons according to their information content. In an unbalanced trial with unequal precision, this makes prediction-error-based reliability measures more appropriate than formulas built on balanced-design assumptions (Piepho and Möhring, 2007; Schmidt et al., 2019).

For SY, PH, NPPP, and SW, GCV and PCV were closely matched, suggesting that the broad phenotypic spread largely reflected genetic differentiation captured by the model. Substantial variability for yield, maturity, and yield components has also been reported in other cowpea germplasm sets, although the magnitude of GCV, PCV, and heritability is necessarily population- and environment-specific (Mofokeng et al., 2020; Owusu et al., 2021). SY was particularly notable in the present panel: despite strong differences in residual precision among years, its GCV and PCV differed by only 0.33 percentage points because persistent genotypic variance was large relative to the residual contribution to an across-year mean.

For SY, Cullis heritability of 0.991 and mean BLUP accuracy of 0.992 indicate very high discriminatory precision within this experiment. Prediction-error-based generalized heritability is especially appropriate in unbalanced breeding trials because it measures the precision of genotype contrasts rather than depending on balanced-design assumptions (Piepho and Möhring, 2007; Schmidt et al., 2019). These estimates should not be read as fixed properties of cowpea or of the individual genotypes. They are conditional on the population, design, sampled environments, and fitted model. What they do show is that this experiment resolved genetic differences unusually well.

The combination of high reliability and large predicted GAM for SY, PH, and NPPP suggests considerable scope for selection, but the result has limits. Cullis or broad-sense heritability does not distinguish additive effects from dominance or epistasis, so high heritability together with high genetic advance cannot be taken as evidence of purely additive gene action. In germplasm screening, the immediate aim is to identify entries that perform consistently well. Whether that superiority is transmitted to segregating progeny must be tested later through crossing, pedigree-informed models, or genomic analysis (Oakey et al., 2007).

For SY, three independent results converge on the same interpretation: the pooled G×Y term was not significant (P = 0.528), REML estimated σ²G×Y/σ²G at only 0.017, and genetic correlations among years were 0.980–0.998. Seasonal conditions therefore changed the scale of yield expression much more than they changed genotype ranking. In mixed-model terms, a strong common genetic signal dominated the covariance structure, with only small year-specific departures (Smith et al., 2001; Malosetti et al., 2013). Gerrano et al. (2020) reported a broadly comparable pattern in a South African dryland cowpea evaluation, where genotype effects were significant but G×E for grain yield was not.

The contrast with multi-location cowpea studies helps put this result in perspective. Kindie et al. (2022) attributed 42.06% of grain-yield variation to GEI across six Ethiopian locations, Kusi et al. (2025) reported a 35.12% GEI contribution across Sudan Savanna and Deciduous Forest environments in Ghana, and Ghazy et al. (2025) found strong environmental control of fresh yield across three Egyptian locations and three seasons. Other studies have likewise documented appreciable re-ranking across contrasting locations or production zones (Horn et al., 2018; Goa et al., 2022; Aleme et al., 2024; Popoola et al., 2024). The much smaller interaction in the present study is therefore credible rather than surprising: three seasons at one site sample temporal variation, but they do not capture the full edaphic and climatic range of a geographically broad target population of environments.

The component traits tell a different story. NSPP had σ²G×Y/σ²G = 0.462 and genetic correlations as low as 0.649, while PL, DTM, and NPBPP also showed substantially more temporal interaction than SY. Stability is therefore trait specific rather than an inherent, across-the-board property of a genotype (Gauch et al., 2008; Malosetti et al., 2013). Shifts in pod number, seed number, and seed size may partly offset one another, allowing final yield to remain comparatively stable even when individual components change rank across seasons (Owusu et al., 2021).

The comparison of residual models is a central methodological result of this study. For SY, the heterogeneous model improved AIC by 50.29 units, driven in large part by the much higher residual variance in 2022 than in 2020 or 2021. Heteroscedastic residuals and environment-specific interaction variances are common in multi-environment datasets, and ignoring them can weaken inference as well as selection decisions (Edwards and Jannink, 2006). Modern mixed-model analyses therefore benefit from modeling genetic covariance and non-genetic heterogeneity together (Smith et al., 2005), preferably at the plot level in a one-stage framework when the data structure allows it (Gogel et al., 2018).

That issue is especially important in an augmented design. Because test entries are unreplicated within a season, the precision of their adjusted values depends heavily on the repeated checks and the local error structure. Residual modeling is therefore integral to the selection problem, not merely a technical refinement: it affects how confidently one entry can be separated from another or from a standard check. Better representation of covariance and precision can materially improve selection accuracy in unbalanced variety trials (Kelly et al., 2007).

BLUP prediction identified a group of entries whose advantage over the best repeated check was substantial rather than marginal. IITA-122, IITA-246, and IITA-153 were predicted to exceed SKUA-WCP-149 by roughly 99–101%, and their prediction accuracies were uniformly high. The practical strength of BLUP in this setting is that it combines shrinkage-adjusted performance estimates with explicit uncertainty. Prediction-error variances show how clearly candidates can be separated instead of relying on rank alone (Piepho et al., 2008). Within the fitted model, the non-overlapping separation between the best-check interval and those of the leading entries provides strong evidence of superiority.

Strong predicted performance at one site should not be mistaken for release readiness. The present trial establishes high genetic merit across three seasons at the test location, but not adaptation across production zones. Multi-environment cowpea studies have repeatedly shown that lines selected in one setting can rerank when agro-ecological conditions change (Goa et al., 2022; Kindie et al., 2022; Popoola et al., 2024; Ghazy et al., 2025; Kusi et al., 2025). IITA-122 and the other leading entries are therefore best viewed as candidates for replicated multi-location testing rather than as final recommendations for broad cultivation.

The wide spread in predicted performance also highlights the value of screening diverse germplasm. Cowpea retains substantial exploitable variation for agronomic and yield-related traits (Boukar et al., 2019; Mofokeng et al., 2020), and recent data from the IITA breeding program show continued genetic progress rather than evidence of a yield plateau (Ongom et al., 2024). Several lines in this panel substantially outperformed the repeated checks under the present conditions and may be valuable either as advanced candidates or as parents in recombination programs.

The contrast between IITA-122 and IITA-84 captures the productivity–stability trade-off particularly well. IITA-122 had the highest mean predicted SY, whereas IITA-84 showed smaller interaction deviations and therefore ranked first for WAASBY while remaining highly productive. Both classical AMMI/GGE approaches and modern mixed-model methods treat mean performance and interaction response as separate dimensions of varietal performance (Gauch et al., 2008). WAASB summarizes the interaction component from BLUPs; WAASBY then places yield and stability on a common rescaled axis using an explicit breeder-defined weighting (Olivoto et al., 2019a; Olivoto et al., 2019b).

The repeated checks make clear why neither productivity nor stability is sufficient on its own. SKUA-WCP-149 was relatively stable (WAASB = 1.88), but its predicted SY was only about half that of the best test entries. Selecting on stability alone would therefore favor consistency without adequate productivity. IITA-52 represents the opposite case: very high predicted yield accompanied by comparatively poor stability. The 65:35 WAASBY weighting offers a transparent compromise between these objectives, but it is not a universal optimum. Different breeding priorities would reasonably produce different rankings among responsive genotypes.

The rank correlations tell the same story from another angle. HMGV, RPGV, and HMRPGV were almost perfectly correlated with mean SY because genotype rankings changed very little across seasons. WAASBY was less tightly aligned with mean yield because it deliberately penalizes instability, whereas stability alone showed a weak negative association with yield. Treating several nearly redundant indices as independent lines of evidence would therefore overstate the amount of distinct information in the analysis (Smith and Cullis, 2018; Olivoto and Lúcio, 2020).

The 13 high-confidence entries are better viewed as a portfolio than as interchangeable winners. A balanced first group includes IITA-84, IITA-122, IITA-95, IITA-257, IITA-264, and IITA-153. IITA-84 is the strongest stability-oriented candidate, while IITA-122 marks the current productivity ceiling. IITA-95 and IITA-257 occupy particularly attractive middle ground, each combining a top-four WAASBY rank with WAASB below 3. This portfolio interpretation is more informative than overemphasizing small differences in ordinal rank because mixed-model selection separates overall performance, stability, and sensitivity into related but distinct decision attributes (Kelly et al., 2007; Smith and Cullis, 2018).

A second group, including IITA-246, IITA-270, IITA-174, and IITA-52, combines very high predicted productivity with larger interaction deviations. These entries may be attractive where maximum yield is the primary objective, but they warrant closer evaluation across additional environments. IITA-52 is the clearest example: it ranked fourth for overall predicted SY but had WAASB = 7.65. IITA-203, IITA-77, and IITA-191 had lower absolute predicted yield than the leading entries, yet still combined clear superiority over the best check with acceptable stability and high conditional selection probability. Maintaining this range of response profiles is more useful for breeding than forcing the entire elite set into a single linear ranking.

The predicted selection differential of 133.76% also requires careful wording. It is the model-based difference between the predicted mean of the selected group and that of the full population, not realized genetic gain in a progeny generation. BLUP-based selection can make phenotypic selection more efficient, but realized progress still depends on inheritance and confirmation in subsequent breeding cycles (Piepho et al., 2008). Even with that qualification, the breeding implication remains strong: the population contains a broad upper tail of high-performing germplasm worth advancing.

Environmental scope is the main limitation of the study. Three seasons at one site provide a meaningful temporal sample, but they do not amount to a geographically representative multi-environment trial. The appropriate language is therefore “temporal stability,” “genotype × year consistency,” and “multi-year performance,” rather than “broad adaptation,” “wide adaptability,” or “climate resilience.” Evidence for broad adaptation requires repeated testing across a target population of environments, coupled with environmental characterization and suitable covariance modeling (Malosetti et al., 2013; Smith et al., 2015). The next validation stage should therefore add contrasting production zones, not simply more years at the same site.

A second limitation is that the analysis is phenotypic and does not separate genotypic variance into additive, dominance, and epistatic components. Pedigree- or genomic-relationship mixed models could partition these effects more explicitly and strengthen inference about parental value (Oakey et al., 2007). Multi-environment genomic prediction could also borrow information across correlated trials when G×E is modeled jointly (Oakey et al., 2016). The single missing SW observation and the unusually large 2022 SY residual variance provide further reasons for replicated follow-up testing. In addition, the available environmental covariates were not sufficient to explain mechanistically either the loss of precision in 2022 or the instability of NSPP. A logical next step is therefore a replicated multi-location trial of the elite portfolio, combined with environmental characterization and, where feasible, genomic relationship information. Factor-analytic mixed models would be well suited to that stage (Smith et al., 2001; Smith and Cullis, 2018).

## Material and methods

### Plant material and study site

The experimental material consisted of 150 cowpea germplasm lines obtained from the International Institute of Tropical Agriculture (IITA), Ibadan, Nigeria, together with four repeated checks: C1, C2, Shalimar Cowpea-1 (SCP-1), and SKUA-WCP-149. C1 and C2 were locally adapted cultivars. SCP-1 was released by Sher-e-Kashmir University of Agricultural Sciences and Technology of Kashmir (SKUAST-K) in 2005, while SKUA-WCP-149 was a locally maintained breeding/check line. The four checks served as adapted references for evaluating the introduced IITA germplasm under temperate conditions.

The following agro-morphological and yield-related traits were recorded:

1. Days to 50% flowering (DTF): DTF was recorded at the plot level as the number of days from sowing until approximately 50% of the plants within a plot had initiated flowering. The date on which this criterion was reached was recorded for each plot and converted to the number of days after sowing.
2. Days to 50% maturity (DTM): DTM was recorded at the plot level as the number of days from sowing until approximately 50% of the pods within a plot had turned yellow. The date on which this criterion was reached was recorded for each plot and expressed as days after sowing.
3. Plant height (PH, cm): Plant height was measured from ground level to the tip of the main axis at the post-flowering stage in 10 randomly selected competitive plants per plot. Border/end plants were excluded from sampling. The measurements of the 10 plants were averaged to obtain one plot-level value.
4. Number of primary branches plant^-^¹ (NPBPP): The number of pod-bearing primary branches arising from the main axis was counted at maturity on the same 10 randomly selected competitive plants per plot. The individual-plant observations were averaged to obtain a single plot-level value.
5. Number of pods plant^-^¹ (NPPP): The total number of pods was counted on the same 10 randomly selected competitive plants per plot. The mean number of pods plant^-^¹ across the 10 sampled plants was calculated and used as the plot-level value.
6. Pod length (PL, cm): Ten pods were selected from each of the 10 sampled plants per plot. Pod length was measured in centimeters for each selected pod. The mean pod length was first calculated for each plant and subsequently averaged across the 10 sampled plants to obtain one plot-level value.
7. Number of seeds pod^-^¹ (NSPP): The number of seeds was counted from the same pods used for pod-length measurement. The mean number of seeds pod^-^¹ was calculated for the sampled plants and then averaged to obtain a single plot-level value.
8. 100-seed weight (SW, g): Following harvesting, clean seeds obtained from the sampled plants within each plot were pooled. A representative subsample of 100 seeds was drawn from the pooled clean seed of each plot, and its weight was recorded in grams. Thus, one 100-seed-weight observation was obtained for each plot.
9. Seed yield plant^-^¹ (SY, gs): Clean seeds obtained from each of the 10 sampled plants per plot were weighed separately. Seed yield plant^-^¹ was calculated for each sampled plant, and the mean of the 10 individual-plant values was used as the plot-level observation.

Field trials were conducted in 2020, 2021, and 2022 at the Experimental Farm of the Division of Genetics and Plant Breeding, Faculty of Agriculture, SKUAST-K, Wadura, Sopore, Jammu and Kashmir, India (34°17′ N, 74°33′ E; approximately 1594 m above mean sea level). Weather during the growing season differed substantially among years. Minimum temperatures ranged from 8.3 to 17.8 °C in 2020, 8.1 to 19.8 °C in 2021, and 10.1 to 20.3 °C in 2022; corresponding maximum temperatures ranged from 22.7 to 33.9 °C, 23.9 to 32.4 °C, and 19.3 to 32.6 °C. Total growing-season rainfall was 146.2 mm in 2020, 463 mm in 2021, and 432 mm in 2022. These contrasts provided the temporal environmental variation needed to assess year-to-year consistency.

### Experimental design and crop management

Each year was evaluated independently using an augmented block design with replicated checks and unreplicated test entries (Federer, 1961). The trial consisted of 10 blocks, each containing 15 test genotypes and the same four checks. This gave 19 plots per block and 190 plots per season: 150 test-entry plots and 40 check plots, with each check represented once in every block (10 replications per season). Across all three years, the experiment generated 570 plot-level records, comprising 450 test-entry plots and 120 check plots. Test genotypes and check positions were re-randomized each year to limit positional confounding while preserving the repeated checks as a common basis for within-year adjustment.

Sowing was carried out during March–April, with harvest during September–October according to genotype maturity and seasonal conditions. Each plot consisted of a single 2-m row, with 80 cm between rows and approximately 20–25 plants maintained per row. Before sowing, farmyard manure was incorporated at 630 kg kanal^-^¹ (approximately 12.45 t ha^-^¹, using 1 kanal = 0.0506 ha). All plots received a basal fertilizer application of 30 kg N, 60 kg P_2_O_5_, and 60 kg K_2_O ha^-^¹. No pre-sowing seed treatment was used. The crop received two to three irrigations during establishment and thereafter depended mainly on prevailing rainfall. Weeds were managed by hand weeding and inter-row cultivation as needed. Genotypes were harvested separately at physiological maturity.

### Phenotypic traits

Phenotypic observations followed the cowpea descriptor framework of the International Board for Plant Genetic Resources (IBPGR) (1983). Nine traits were recorded: days to 50% flowering (DTF), days to 50% maturity (DTM), plant height (PH, cm), number of primary branches per plant (NPBPP), number of pods per plant (NPPP), pod length (PL, cm), number of seeds per pod (NSPP), 100-seed weight (SW, g), and seed yield per plant (SY, g plant^-^¹).

### Statistical analysis

The analysis combined year-specific, design-based assessment with multi-year mixed-model prediction. Each year contributed 190 plot-level observations (150 test-entry plots and 40 check plots), giving n = 570 observations across the three seasons; one 100-seed-weight value was missing, so n = 569 for that trait. The across-year models included 150 test entries and four repeated checks. Unless noted otherwise, α = 0.05. F tests and likelihood-ratio tests used their standard upper-tail reference distributions.

Mixed-model analyses, including REML estimation and BLUP-based prediction, were performed using the sommer package (version 4.4.5). Multi-environment analysis and stability/productivity indices, including WAASB, WAASBY, HMGV, RPGV and HMRPGV, were performed using metan (version 1.19.0). The car package (version 3.1-5) was used for relevant statistical tests, while data manipulation and preparation were conducted using dplyr (version 1.2.1) and tidyr (version 1.3.2). Graphical visualization was performed using ggplot2 (version 4.0.3).

### Year-wise augmented-block analysis

Within each year, genotype effects were tested after adjustment for block effects using the augmented-block model shown below. With 150 test entries and four repeated checks, 10 blocks, the F tests used 153 numerator and 27 denominator degrees of freedom; these values generated the exact P values in **Table 1**.


$${\text{Y}}_{\text{ij}}=\mu +{\text{B}}_{\text{j}}+{\text{G}}_{\text{i}}+\epsilon_{\text{ij}},$$

where Y_ij_ is the observed phenotypic value of genotype i in block j, μ is the overall mean, B_j_ is the block effect, G_i_ is the genotype effect, and ϵ_ij_ is the residual. Because test entries were unreplicated within a season, the repeated checks supplied the experimental-error basis for adjustment and inference. Residual coefficients of variation (CV%) were used to describe year-specific field precision.

### Combined fixed-effect analysis of seed yield

For SY, we also fitted a combined fixed-effect model as a conventional omnibus test of year, genotype, and G×Y effects:


$${\text{Y}}_{\text{ijk}}=\mu +{\text{G}}_{\text{i}}+{\text{Y}}_{\text{j}}+{\left(\text{GY}\right)}_{\text{ij}}+{\text{B}}_{\text{k}}\left({\text{Y}}_{\text{j}}\right)+\epsilon_{\text{ijk}},$$

where Y_j_ is the year effect, (GY)_ij_ is the genotype × year interaction, and B_k_(Y_j_) denotes block k nested within year j. This model provided a complementary mean-square summary; variance-component and covariance models remained the basis for prediction.

### REML variance components, final mixed model and BLUPs

The model statement below assumes that block nested within year was included in the fitted multi-year REML models as a fixed design effect.

The complete multi-year REML/BLUP model was specified as:


$${\text{Y}}_{\text{ijk}}=\mu +{\text{Y}}_{\text{j}}+{\text{B}}_{\text{k}}\left({\text{Y}}_{\text{j}}\right)+{\text{G}}_{\text{i}}+{\left(\text{GY}\right)}_{\text{ij}}+\epsilon_{\text{ijk}},$$

where Y_ijk_ is the plot-level observation for genotype i in year j and block k; μ is the overall mean; Y_j_ is the fixed year effect; B_k_(Y_j_) is block k nested within year j and is included as a fixed design effect to retain the augmented-block adjustment; G_i_ is the random genotype effect, G_i_ ~ N(0, σ²G); (GY)_ij_ is the random genotype × year effect, (GY)_ij_ ~ N(0, σ²G×Y); and ϵ_ijk_ is the residual. For traits for which residual heterogeneity was selected, ϵ_ijk_ ~ N(0, σ²e,j), allowing a separate residual variance for each year; otherwise a common residual variance was used. Variance components were estimated by restricted maximum likelihood (REML) (Patterson and Thompson, 1971), and genotype-specific genetic merit was predicted by best linear unbiased prediction (BLUP) (Henderson, 1975; Piepho et al., 2008). The size of temporal interaction relative to persistent genotypic variation was expressed as σ²G×Y/σ²G, and consistency as σ²G/(σ²G + σ²G×Y).

For the across-year genetic-covariance analysis, the genotype effects across the three years were additionally parameterized as a three-element random vector and fitted with either compound-symmetry or unstructured genetic covariance; the same fixed year and block(year) design terms and the selected residual-variance structure were retained.

### Residual heterogeneity and model selection

Equality of residual variance among years was first screened with the Brown–Forsythe test (Brown and Forsythe, 1974). For each trait, we compared a model with a common residual variance against one that allowed a separate residual variance for each year. Fixed-effect specifications were identical in the competing models, which were compared using Akaike’s information criterion (AIC) and likelihood-ratio tests. A heterogeneous structure was retained when it materially improved model fit. In practical terms, this allowed a low-precision season to contribute less information to pooled prediction than a high-precision season.

### Genetic and phenotypic variability, heritability and genetic advance

Genotypic and phenotypic coefficients of variation were expressed on the trait-mean scale:


$$\text{GCV}\left(\%\right)=100\times \surd \sigma^{2}\text{G}/\bar{x};\;\text{PCV}\left(\%\right)=100\times \surd \sigma^{2}\text{P}/\bar{x},$$

where $\bar{x}$ is the overall trait mean. The term σ²P represents the model-implied variance of an across-year genotype mean, incorporating persistent genotypic variance together with appropriately averaged G×Y and residual contributions. Cullis heritability was calculated from the mean prediction-error variance of pairwise genotype differences (PEVΔ):


$${\text{H}}^{2}\text{Cullis}=1-\text{PEV}\Delta /\left(2\sigma^{2}\text{G}\right).$$

Genotype-specific BLUP accuracy was derived from prediction-error variance relative to σ^2^G, and mean accuracy was summarized for each trait. Expected genetic advance at 5% selection intensity used i = 2.063 and the standard expression GA = i × σP × H^2^; genetic advance as a percentage of the mean was calculated as $\text{GAM}=100\times \text{GA}/\bar{x}$ (Johnson et al., 1955). These estimates describe predicted response under the fitted model and are not equivalent to realized genetic gain in a subsequent breeding generation.

### Across-year genetic covariance and rank preservation

To assess preservation of genotype ranking across seasons, we fitted a three-year unstructured genetic covariance model and compared it with compound symmetry using AIC. Pairwise genetic correlations were calculated as


$$\text{rG}\left(\text{jk}\right)=\sigma \text{G}\left(\text{jk}\right)/\surd \left[\sigma^{2}\text{G}\left(\text{j}\right)\sigma^{2}\text{G}\left(\text{k}\right)\right].$$

Values close to one indicate strong preservation of genotype ranking; lower correlations imply greater temporal re-ranking. We interpreted correlation magnitude together with covariance-model fit, because a more flexible covariance structure can improve fit even when all correlations remain high.

### Productivity, stability and integrated selection

Overall and year-specific adjusted genotype predictions were obtained from the final REML models. Approximate 95% prediction intervals were constructed from genotype-specific prediction standard errors. Superiority over the best repeated check was calculated as 100 × (predicted genotype value − predicted best-check value)/predicted best-check value. Reaction norms were used to display the seasonal trajectories of leading genotypes.

Temporal stability of SY was summarized with WAASB, which is based on the absolute weighted scores from singular-value decomposition of the BLUP G×Y interaction matrix; smaller WAASB values reflect smaller interaction deviations (Olivoto et al., 2019a). WAASBY combined rescaled yield performance and stability, assigning 65% weight to yield and 35% to stability, with both components oriented so that larger scores were favorable (Olivoto et al., 2019a; Olivoto et al., 2019b). HMGV, RPGV, and HMRPGV were also calculated as BLUP-based productivity/stability summaries, and Spearman rank correlations were used to assess redundancy among the indices (Olivoto and Lúcio, 2020).

A high-confidence elite set was defined for decision support using three simultaneous criteria: top-decile overall predicted SY, top-decile WAASBY, and a conditional probability of at least 0.80 of belonging to the yield top 10%. The probability was calculated under a normal approximation to each genotype’s fitted prediction distribution relative to the empirical top-decile yield cutoff. Because this is a model-based decision quantity rather than a biological trait or realized response, it was considered alongside prediction intervals, accuracy, and stability.

### Plant research compliance

The experiments used cultivated cowpea germplasm obtained from the International Institute of Tropical Agriculture, Ibadan, Nigeria. The study was conducted in accordance with relevant institutional, national, and international guidelines for research involving plants.

## Data Availability

The original contributions presented in the study are included in the article/**Supplementary Material**. Further inquiries can be directed to the corresponding author.
