# Supplementary material for: Precision-weighted mixed models identify high-yielding and temporally stable cowpea germplasm across three growing seasons
Source: Front Plant Sci. 2026 Oct 7;17:1926051. doi: 10.3389/fpls.2026.1926051 (PMC13683631; doi:10.3389/fpls.2026.1926051)
Supplement: Supplementary file 1 [file SupplementaryFile1.docx]

# Supplementary material

**Supplementary Figure S1.** Relationship between Cullis heritability and genetic advance as percentage of the mean (GAM) at 5% selection intensity. Dashed lines indicate the medians of the observed trait distributions and are descriptive guides rather than inferential thresholds. SY combines the highest expected proportional gain with very high prediction reliability.

**Trait abbreviations used figure**

DTF, days to flowering; DTM, days to maturity; PH, plant height; NPBPP, number of primary branches per plant; NPPP, number of pods per plant; PL, pod length; NSPP, number of seeds per pod; SW, 100-seed weight; SY, seed yield.
