# Supplementary material for: Precision-weighted mixed models identify high-yielding and temporally stable cowpea germplasm across three growing seasons
Source: Front Plant Sci. 2026 Oct 7;17:1926051. doi: 10.3389/fpls.2026.1926051 (PMC13683631; doi:10.3389/fpls.2026.1926051)
Supplement: Supplementary file 2 [file SupplementaryFile2.docx]

# Supplementary material

**Supplementary Figure S2.** Integrated productivity–stability ranking based on WAASBY (65% weight on performance and 35% on stability). Higher values indicate a more favorable combined profile. IITA-84 ranks above the maximum-yield genotype IITA-122 because of its superior temporal stability.
