# Supplementary material for: Precision-weighted mixed models identify high-yielding and temporally stable cowpea germplasm across three growing seasons
Source: Front Plant Sci. 2026 Oct 7;17:1926051. doi: 10.3389/fpls.2026.1926051 (PMC13683631; doi:10.3389/fpls.2026.1926051)
Supplement: Supplementary file 3 [file SupplementaryFile3.docx]

# Supplementary material

**Supplementary Figure S3.** Integrated percentile-based decision profile of high-confidence elite genotypes and the best check. Metrics were transformed to a common 0–100 percentile scale, with higher values favorable; WAASB was reverse-scored so that greater stability receives a higher percentile. The figure exposes distinct routes to elite status, from balanced productivity–stability to yield-dominant selection.
