# Supplementary material for: Precision-weighted mixed models identify high-yielding and temporally stable cowpea germplasm across three growing seasons
Source: Front Plant Sci. 2026 Oct 7;17:1926051. doi: 10.3389/fpls.2026.1926051 (PMC13683631; doi:10.3389/fpls.2026.1926051)
Supplement: Supplementary file 4 [file SupplementaryFile4.docx]

# Supplementary material

**Supplementary Figure S4.** Year-specific residual variance for seed yield from the selected heterogeneous mixed model. The sharp increase in 2022 explains the reduced year-specific F statistic and justifies unequal residual weighting across seasons.
