# Supplementary material for: Precision-weighted mixed models identify high-yielding and temporally stable cowpea germplasm across three growing seasons
Source: Front Plant Sci. 2026 Oct 7;17:1926051. doi: 10.3389/fpls.2026.1926051 (PMC13683631; doi:10.3389/fpls.2026.1926051)
Supplement: Supplementary file 5 [file Table1.docx]

**Supplementary material**

**Supplementary Table S1.** Residual-variance diagnostics and year-specific error variances.

| **Trait** | **BF P** | **ΔAIC hom–het** | **LRT P** | **Selected residual** | **σ²e 2020** | **σ²e 2021** | **σ²e 2022** |
| --- | --- | --- | --- | --- | --- | --- | --- |
| DTF | 1.51e-09 | 48.51 | 3.95e-12 | Heterogeneous | 7.25 | 1.39 | 1.09 |
| DTM | 0.00 | 17.40 | 2.25e-05 | Heterogeneous | 9.81 | 12.91 | 3.01 |
| PH | 0.48 | -0.15 | 0.15 | Homogeneous | 4.58 | 4.58 | 4.58 |
| NPBPP | 0.11 | -3.12 | 0.64 | Homogeneous | 0.78 | 0.78 | 0.78 |
| NPPP | 0.29 | 3.18 | 0.03 | Heterogeneous | 2.56 | 0.94 | 1.42 |
| PL | 0.30 | 0.94 | 0.08 | Homogeneous | 0.76 | 0.76 | 0.76 |
| NSPP | 0.30 | -2.85 | 0.56 | Homogeneous | 0.73 | 0.73 | 0.73 |
| SW | 0.80 | -2.12 | 0.39 | Homogeneous | 0.90 | 0.90 | 0.90 |
| SY | 0.01 | 50.29 | 1.62e-12 | Heterogeneous | 7.92 | 5.90 | 56.55 |

*BF = Brown–Forsythe test. Positive ΔAIC indicates improved fit of the heterogeneous residual model.*

**Trait abbreviations used in table**

DTF, days to flowering; DTM, days to maturity; PH, plant height; NPBPP, number of primary branches per plant; NPPP, number of pods per plant; PL, pod length; NSPP, number of seeds per pod; SW, 100-seed weight; SY, seed yield
