# Supplementary material for: Precision-weighted mixed models identify high-yielding and temporally stable cowpea germplasm across three growing seasons
Source: Front Plant Sci. 2026 Oct 7;17:1926051. doi: 10.3389/fpls.2026.1926051 (PMC13683631; doi:10.3389/fpls.2026.1926051)
Supplement: Supplementary file 6 [file Table2.docx]

# Supplementary material

**Supplementary Table S2.** Combined fixed-effect ANOVA for seed yield across three growing seasons.

| **Source** | **df** | **SS** | **MS** | **F** | **P value** | **Error CV %** |
| --- | --- | --- | --- | --- | --- | --- |
| Year | 2 | 588.01 | 294.01 | 7.11 | 0.00 | 10.32 |
| Block(Year) | 27 | 8.94e+04 | 3311.27 | 80.08 | 1.17e-47 | 10.32 |
| Genotype | 153 | 5.99e+05 | 3912.75 | 94.63 | 9.71e-61 | 10.32 |
| Genotype×Year | 306 | 1.26e+04 | 41.08 | 0.99 | 0.53 | 10.32 |

*The genotype × year term was not significant (P = 0.528), consistent with the very small mixed-model interaction variance relative to σ²G.*
