# Supplementary material for: Precision-weighted mixed models identify high-yielding and temporally stable cowpea germplasm across three growing seasons
Source: Front Plant Sci. 2026 Oct 7;17:1926051. doi: 10.3389/fpls.2026.1926051 (PMC13683631; doi:10.3389/fpls.2026.1926051)
Supplement: Supplementary file 7 [file Table3.docx]

# Supplementary material

**Supplementary Table S3.** Leading seed-yield genotypes from mixed-model prediction and temporal-stability analysis.

| **Genotype** | **Overall SY** | **95% PI** | **Accuracy** | **WAASB** | **WAASBY** | **HMRPGV** | **Yield rank** | **WAASBY rank** | **Superiority vs best check %** | **Top-10% prob.** |
| --- | --- | --- | --- | --- | --- | --- | --- | --- | --- | --- |
| IITA-122 | 156.94 | 149.32–164.57 | 0.992 | 3.59 | 92.19 | 2.466 | 1 | 2 | 100.60 | 1.000 |
| IITA-246 | 156.20 | 148.57–163.82 | 0.992 | 5.56 | 87.29 | 2.453 | 2 | 8 | 99.64 | 1.000 |
| IITA-153 | 155.21 | 147.58–162.83 | 0.992 | 4.15 | 90.10 | 2.438 | 3 | 6 | 98.38 | 1.000 |
| IITA-52 | 155.09 | 147.46–162.71 | 0.992 | 7.65 | 81.97 | 2.435 | 4 | 16 | 98.23 | 1.000 |
| IITA-270 | 154.91 | 147.29–162.53 | 0.992 | 5.31 | 87.29 | 2.433 | 5 | 9 | 98.00 | 1.000 |
| IITA-222 | 154.10 | 146.47–161.72 | 0.992 | 8.36 | 79.87 | 2.418 | 6 | 18 | 96.96 | 1.000 |
| IITA-174 | 152.11 | 144.49–159.73 | 0.992 | 5.94 | 84.55 | 2.389 | 7 | 12 | 94.42 | 1.000 |
| IITA-95 | 152.02 | 144.39–159.64 | 0.992 | 2.87 | 91.61 | 2.389 | 8 | 3 | 94.30 | 1.000 |
| IITA-189 | 150.96 | 143.34–158.58 | 0.992 | 9.92 | 74.83 | 2.367 | 9 | 20 | 92.95 | 1.000 |
| IITA-264 | 150.74 | 143.11–158.36 | 0.992 | 3.14 | 90.40 | 2.368 | 10 | 5 | 92.67 | 1.000 |
| IITA-84 | 150.56 | 142.94–158.18 | 0.992 | 1.61 | 93.85 | 2.366 | 11 | 1 | 92.44 | 1.000 |
| IITA-257 | 149.95 | 142.32–157.57 | 0.992 | 2.65 | 91.16 | 2.356 | 12 | 4 | 91.66 | 1.000 |
| IITA-77 | 147.63 | 140.00–155.25 | 0.992 | 5.52 | 83.48 | 2.318 | 13 | 14 | 88.69 | 1.000 |
| IITA-191 | 143.74 | 136.12–151.37 | 0.992 | 5.08 | 82.73 | 2.258 | 14 | 15 | 83.73 | 0.982 |
| IITA-203 | 142.42 | 134.80–150.04 | 0.992 | 3.46 | 85.87 | 2.237 | 15 | 10 | 82.04 | 0.954 |
| SKUA-WCP-149 | 78.24 | 71.98–84.49 | 0.994 | 1.88 | 60.22 | 1.229 | 37 | 34 | 0.00 | 0.000 |

*Lower WAASB denotes greater stability; WAASBY combines 65% performance and 35% stability. PI = prediction interval*

**Trait abbreviations used in table**

WAASB, weighted average of absolute scores from BLUP interaction effects; WAASBY, WAASB-derived superiority index integrating performance and stability; HMGV, harmonic mean of genotypic values; RPGV, relative performance of genotypic values; HMRPGV, harmonic mean of relative performance of genotypic values.
