# Supplementary material for: Precision-weighted mixed models identify high-yielding and temporally stable cowpea germplasm across three growing seasons
Source: Front Plant Sci. 2026 Oct 7;17:1926051. doi: 10.3389/fpls.2026.1926051 (PMC13683631; doi:10.3389/fpls.2026.1926051)
Supplement: Supplementary file 8 [file Table4.docx]

| **Metric** | **Value** |
| --- | --- |
| Population predicted mean | 63.64 |
| Top-10% WAASBY selected N | 16 |
| Selected predicted mean | 148.76 |
| Absolute selection differential | 85.12 |
| Selection differential (%) | 133.76 |
| Best check | SKUA-WCP-149 |
| Best-check predicted mean | 78.24 |
| Selected mean superiority over best check (%) | 90.14 |

**Supplementary material**

**Supplementary Table S4.** Selection response and robustness of elite-genotype identification.

*Selection differentials are predicted contrasts under the fitted model and should not be described as realized genetic gain*
